# Supplementary material for: Severity of Omicron Subvariants and Vaccine Impact in Catalonia, Spain
Source: Vaccines (Basel). 2024 Apr 27;12(5):466. doi: 10.3390/vaccines12050466 (PMC11125683; doi:10.3390/vaccines12050466)
Supplement: Supplementary file 1 [file vaccines-12-00466-s001.zip › Suppl_Mat_Text_Vaccines_DEF.pdf]

Supplementary Material on *Severity of Omicron Subvariants and Vaccine Impact in Catalonia, Spain*

Víctor López de Rioja, Luca Basile, Aida Perramon-Malavez, Érica Martínez-Solanas, Daniel López, Sergio Medina Maestro, Ermengol Coma, Francesc Fina, Clara Prats, Jacobo Mendioroz Peña, and Enric Alvarez-Lacalle.

Suppl. Mat. Text S1. COVID-19 Variant Sampling in Catalonia 2

Suppl. Mat. Text S2. ASPCAT follow-ups vs total registered cases 3

Suppl. Mat. Text S3. Evaluating the fluctuations in cases, hospitalizations, and deaths across variants 5

Suppl. Mat. Text S4. Methodology in the severity metrics by vaccination status 7

Suppl. Mat. Text S5. Severity of outcomes across age and vaccination status: expanded explanation and extended results 11

Suppl. Mat. Text S6. Analysis of vaccine impact across age cohorts in 2022 and detailed mortality analysis data including deaths due to and with COVID-19 15

Suppl. Mat. Text S1. COVID-19 Variant Sampling in Catalonia

In the surveillance of COVID-19 variants within Catalonia, the sampling strategy incorporates both random and targeted approaches to ensure a comprehensive analysis of the spread and evolution of the virus.

Approximately 70-80% of the samples are randomly collected from primary care centres and hospitals to ensure a broad representation of the population. The remaining 20-30% are selectively chosen for reasons such as vaccination failure or outbreaks, but also severe cases in younger individuals, suspected reinfection, and high transmissibility, often to identify new variants in the region. For outbreak studies, it is considered sufficient to sequence 10% of the samples, assuming that linked cases are caused by the same variant. This mix of random and targeted sampling helps to provide a comprehensive view of variant spread, addressing both general incidence and specific public health concerns.

Suppl. Mat. Text S2. ASPCAT follow-ups vs total registered cases

Comparison between the number of cases registered by ASPCAT and the number of surveys conducted on a weekly basis.

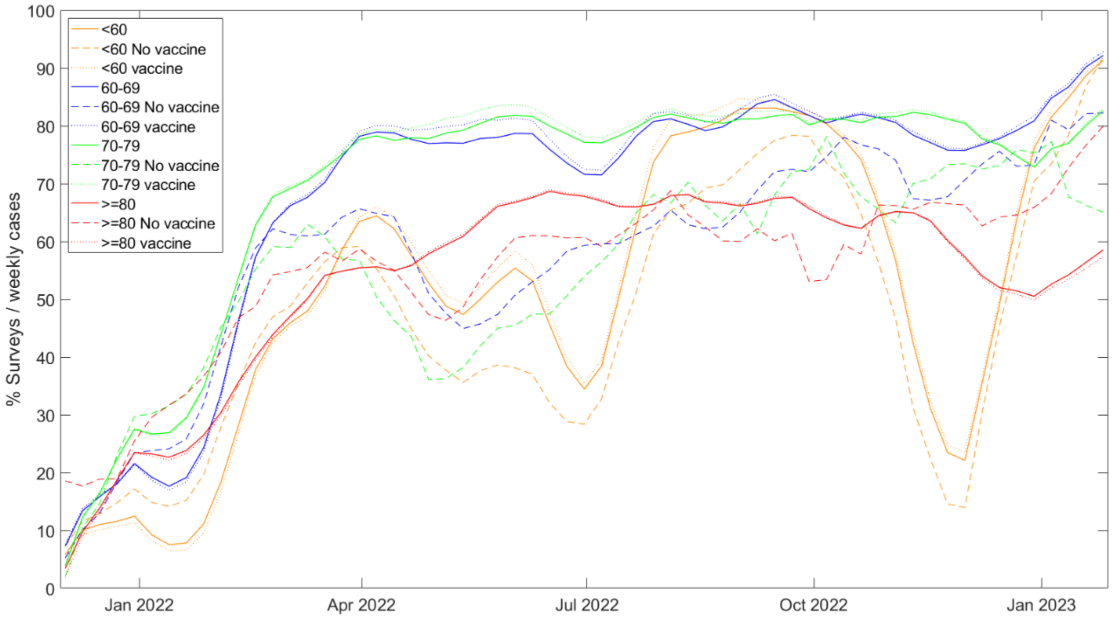

Figure S1 Smoothed percentage comparison between survey responses and total cases from ASPCAT across four age groups and their vaccination status.

Comparison of metrics in the study: cases, hospitalizations, and deaths by/with COVID-19 for the database with surveys conducted by ASPCAT (right) and for the total ASPCAT database (left). Note that at the beginning of 2022 (BA.1 variant and the highest peak of cases in the history of Catalonia), it is not possible to cover all patients. There was a decision to prioritize the most vulnerable, conducting thorough follow-ups mainly on people over 60 years old.

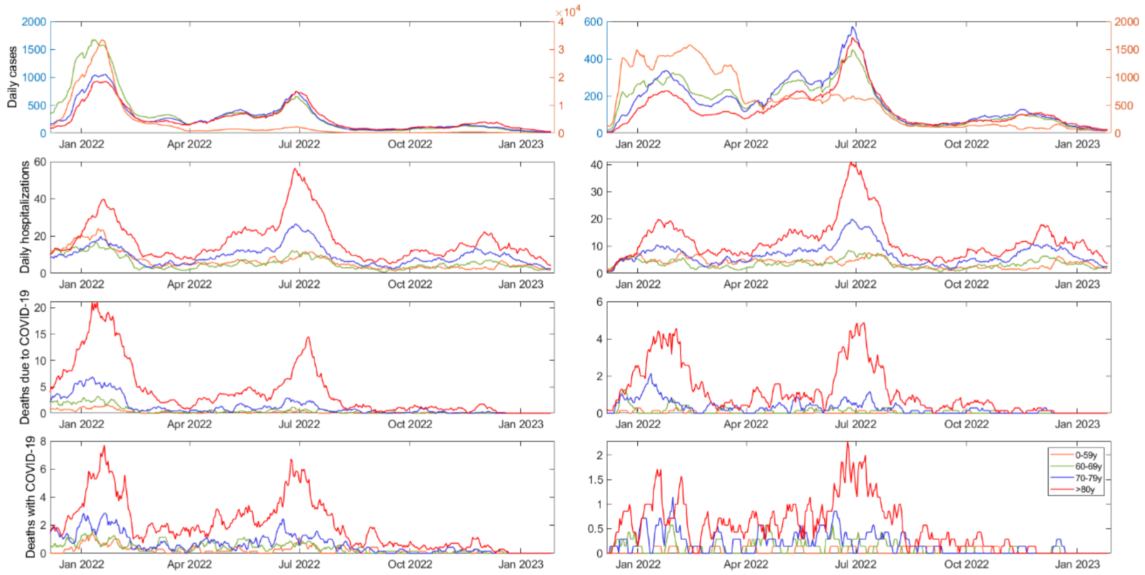

Figure S2 Comparison of the entire ASPCAT database (left column) and only patients with a completed survey (right column). Cases, new hospitalizations, deaths due to and with COVID-19 are shown on a daily basis. All dates are referenced to the day of diagnosis.

Figure S3 illustrates the temporal relationship between the emergence of different omicron subvariants and associated severity metrics within the Catalan population, segmented by age groups. Using data from the ASPCAT database, which contains 415,629 COVID-19 entries, we examine daily cases, hospitalizations, and deaths. The emerging patterns of subvariants BA.1, BA.2, BA.5 and BQ.1 are delineated by vertical lines - dashed, solid and again dashed - indicating the 10%, 50% and 90% prevalence thresholds, respectively. These visual boundaries provide context for the epidemiological impact of each variant at different stages of prevalence.

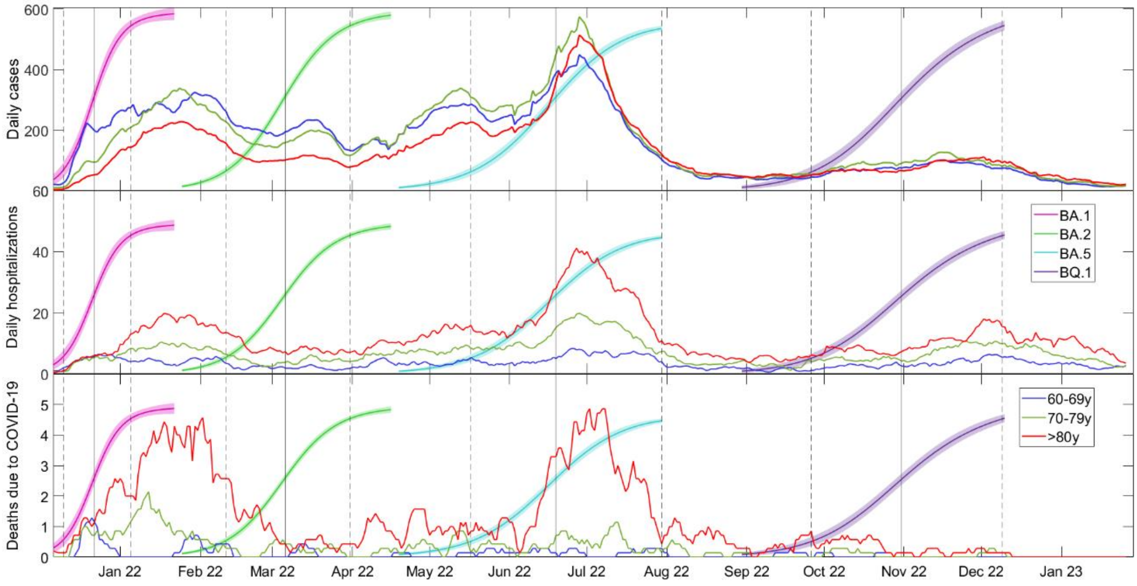

Figure S3 Relationship between the emergence of different Omicron variants and severity metrics in Catalonia based on cases tracked by ASPCAT (415,629 COVID-19 data entries). The vertical lines—dashed, solid, and again dashed—represent the 10%, 50%, and 90% prevalence levels of the four variants: BA.1, BA.2, BA.5, and BQ.1.

Suppl. Mat. Text S3. Evaluating the fluctuations in cases, hospitalizations, and deaths across variants

To deeply analyse the data in 3.1, we examine the rate of change of various metrics over time to identify periods of increase and decrease. **Figure S4** displays the rate of change of the metrics shown in **Figure S3** (cases, hospitalizations, and deaths) averaged over seven days. The background is color-coded to indicate an increase (green) or decrease (red) in each metric. Although **Figure S4** depicts three age groups, the red/green colour scheme specifically refers to patients over the age of 80.

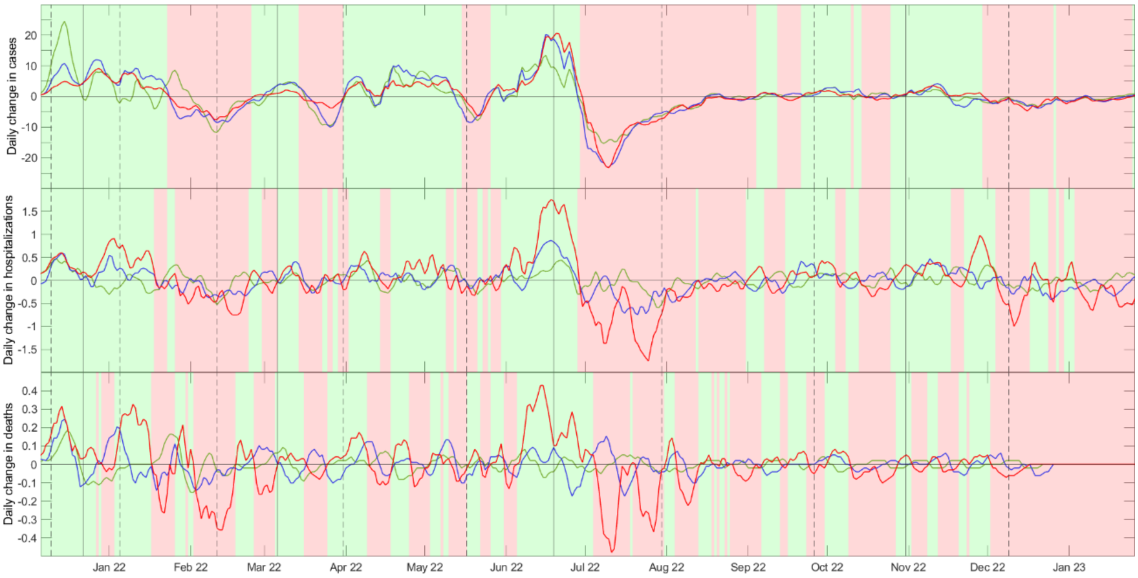

Figure S4 Daily changes (derivative) in cases, hospitalizations, and deaths (due to COVID-19) for individuals older than 80 years.

Importantly, we observe a consistent increase in all three metrics for both the BA.1 and BA.5 variants when each represents approximately 50% of the circulating viral landscape in the region. This suggests that the population that has not yet been immunized against these emerging lineages is experiencing a greater impact, particularly among the elderly. The BA.2 variant appears to be quantitatively less severe, possibly due to the overshadowing effect of its predecessor, BA.1, and the rapid transition from BA.1 to BA.2 that occurred over a period of only two months. Interestingly, despite the lower overall numbers compared to BA.1 and BA.5, the majority of hospitalizations and deaths occur during the months when BA.2 is dominant, exceeding those recorded during its emergence. The BQ.1 lineage exhibits not small increases in cases and hospitalizations after becoming dominant, between 50-80% of their entrance. This trend is observed in all age groups but is more pronounced in older individuals. Interestingly, as the next dominant variant, XBB.1.5, is not expected to establish dominance in Catalonia until the end of February 2023, the remainder of BQ.1's dominance is characterized by either a decrease or stabilization at very low levels in the number of cases, hospitalizations, and deaths in all age groups.

Table S1 aligns the results of **Figure S4**, which focuses on individuals over the age of 80, with the two periods defined in **Section 3.1:** emergence and dominance. These results underscore the increased risk during the emergence periods of the variants, which requires increased vigilance. The average increases in cases, hospitalizations, and deaths are generally higher during the emergence period than during the dominance period. An exception to this trend is the Omicron

BA.2 lineage, possibly due to the high number of cases associated with its predecessor, BA.1. In addition, there is potential data overlap exists with the early presence (0-10%) of BA.5, which may result in the misattribution of cases or hospitalizations initially attributed to BA.2, as evidenced by the peak in daily cases or hospitalizations just before BA.5 crosses the 10% threshold, indicated by the vertical dashed line in **Figure 3**.

Table S1 Table of the average increase (or decrease) of the cases, hospitalizations, and deaths for the two periods of emergence and dominance of the study variants BA.1, BA.2, BA.5, and BQ.1 on individuals over the age of 80.

	Average increase (positive) or decrease (negative) of								
	Cases/day			Hospitalizations/day			Deaths/day		
	**60-69**	**70-79**	**>80**	**60-69**	**70-79**	**>80**	**60-69**	**70-79**	**>80**
BA.1 emergence	8.7	8.8	6.15	0.1	0.3	0.23	0.00	0.07	0.04
BA.1 dominance	1.8	0.9	1.56	0.0	-0.1	0.22	0.03	-0.06	0.03
BA.2 emergence	-3.1	-3.5	-1.63	-0.1	-0.1	-0.22	0.00	0.00	-0.04
BA.2 dominance	-0.5	1.2	0.52	0.0	0.0	-0.13	0.00	0.00	0.00
BA.5 emergence	-0.2	0.0	0.13	0.1	0.1	-0.03	0.01	0.00	0.00
BA.5 dominance	-2.1	-2.6	-1.64	0.0	-0.2	-0.07	-0.02	0.00	0.00
BQ.1 emergence	1.1	1.3	1.19	0.0	0.2	0.28	0.00	0.00	0.00
BQ.1 dominance	-1.9	-2.3	-2.21	-0.1	-0.3	-0.52	0.00	0.00	-0.02

Suppl. Mat. Text S4. Methodology in the severity metrics by vaccination status

This section of the supplementary material details the steps followed to obtain **Figure 2** of the main manuscript, that is, the percentages of hospitalization and death with respect to cases and hospitalizations, focusing exclusively on the data for the age group over 80 years old. The other age groups were analysed in a similar manner.

In the separately attached spreadsheet files (**Suppl. Mat. Tables S3-S5**), we can find the number of cases, hospitalizations, and deaths week by week, organized according to the time since their last COVID-19 vaccination (always either full vaccination schedule or first or second booster dose). Taking into account the weeks of dominance of each variant (as defined in the main manuscript), we can sum the entries from the ASPCAT database to obtain **Table S2**:

Table S2 Sample of cases, hospitalizations, total deaths, and in-hospital deaths for the population aged 80 and over in Catalonia, divided by time intervals since the last vaccine (complete schedule or booster dose) and by period of Omicron variant dominance.

	1-3 months	4-6 months	7-9 months	10+ months	NO vaccination
	Cases				
BA.1	6257	1393	591	635	682
BA.2	691	8242	1517	667	365
BA.5	159	1601	17780	4023	802
BQ.1	2399	215	74	5125	367
	Hospitalizations				
BA.1	388	89	86	76	147
BA.2	43	577	96	79	70
BA.5	15	117	1439	365	115
BQ.1	337	48	12	726	84
	Deaths (due to COVID-19)				
BA.1	58	20	14	19	40
BA.2	2	43	10	2	8
BA.5	4	6	119	37	16
BQ.1	4	0	0	16	1
	in-hospital deaths (due to COVID-19)				
BA.1	31	11	12	10	26
BA.2	0	26	5	0	5
BA.5	3	2	49	15	9
BQ.1	2	0	0	9	1

<u>Rate ratio</u>

Our database of the Public Health Agency of Catalonia (ASPCAT), records and tracks the evolution of the disease for the most vulnerable confirmed COVID-19 patients. Therefore, it is not quite correct to speak of a *risk ratio* or to calculate the *hazard ratio*, which are common in other

types of studies with a control group, usually based on the TNCC methodology. For this reason, we defined a rate ratio (RR), as other studies have done before[1,2]:

$$RR_{Hosp} = \frac{\frac{\text{hospitalized by COVID, vaccinated}}{\text{confirmed cases of COVID, vaccinated}}}{\frac{\text{hospitalized by COVID, unvaccinated}}{\text{confirmed cases of COVID, unvaccinated}}},$$

$$RR_{Death} = \frac{\frac{\text{death due to COVID, vaccinated}}{\text{confirmed cases of COVID, vaccinated}}}{\frac{\text{death due to COVID, unvaccinated}}{\text{confirmed cases of COVID, unvaccinated}}},$$

$$RR_{in-Hosp\ Death} = \frac{\frac{\text{in hospital death due to COVID, vaccinated}}{\text{hospitalized by COVID, vaccinated}}}{\frac{\text{in hospital death due to COVID, unvaccinated}}{\text{hospitalized by COVID, unvaccinated}}}.$$

In fact, we could say that the $RR_{in-Hosp\ Death}$ is the same as the risk ratio calculated by other studies.

Binomial distribution for confidence intervals

For these RR values, we employed a binomial distribution to model the different rates, as each COVID-19 case independently resulted in one of two categorical outcomes: hospitalization or non-hospitalization; death and in-hospital death or survival. In our case, to follow the same method as below with the Fisher's test, we employ the most widely used exact interval in the literature, the Clopper-Pearson interval, introduced by Clopper & Pearson in 1934. The interval for a proportion p contains all the values of p that aren't rejected by the test at confidence level α, in our case $\alpha = 0.05$. Given an observation X, the lower and upper limit are given by:

$$\sum_{k=X}^{n} \binom{n}{k} p_L^k (1 - p_L)^{n-k} = \alpha/2,$$

$$\sum_{k=0}^{X} \binom{n}{k} p_U^k (1 - p_U)^{n-k} = \alpha/2.$$

These intervals were calculated for this method with the MATLAB function `binofit(x,n)`, where x is the number of observed successes and n is the number of independent trials.

Table S3 shows all the rate ratio values for the +80-age cohort along with the confidence intervals in parentheses calculated as just explained. Red shades, which appear predominantly on the right side of the table, indicate greater severity and are indicative of unvaccinated individuals or those with outdated vaccinations. In contrast, green shades on the left indicate milder results. However, there are some values that stand out, for example because of high severity in places where we would not expect to find them, such as recently vaccinated people. This is understandable because the number of cases (hospitalizations or deaths) may be very small, with very wide confidence intervals, and may not be directly comparable to the rest of the results from the same group. To understand this, we calculated their p-values.

---

[1] Marrone, G.; et al. *Euro Surveill*. **2022**, *27(7)*, 2200060. https://doi.org/10.2807/1560-7917.ES.2022.27.7.2200060
[2] Catala, M.; et al. *Front. Public Health*. **2022**, *10*, 961030. https://doi.org/10.3389/fpubh.2022.961030

Table S3 Percentage relationship of cases, hospitalizations, and COVID-19 deaths according to the periods of each study variant for different vaccination statuses of individuals older than 80 years in Catalonia. The rest of the age groups can be found in **Suppl. Mat. Tables S3, S4, and S5**.

	1-3 months	4-6 months	7-9 months	10+ months	NO vaccination
	% Hosp vs cases (95% CI)				
BA.1	6.20 (5.62 – 6.83)	6.39 (5.16 – 7.80)	14.55 (11.81 – 17.66)	11.97 (9.55 – 14.75)	21.55 (18.52 – 24.83)
BA.2	6.22 (4.54 – 8.29)	7.00 (6.46 – 7.57)	6.33 (5.16 – 7.67)	11.84 (9.49 – 14.54)	19.18 (15.27 – 23.60)
BA.5	9.43 (5.38 – 15.08)	7.31 (6.08 – 8.69)	8.09 (7.70 – 8.50)	9.07 (8.20 – 10.00)	14.34 (11.99 – 16.96)
BQ.1	14.05 (12.68 – 15.50)	22.33 (16.94 – 28.49)	16.22 (8.67 – 24.61)	14.17 (13.22 – 15.15)	22.89 (18.69 – 27.53)
	% Deaths vs cases (90% CI)				
BA.1	0.93 (0.70 – 1.20)	1.44 (0.88 – 2.21)	2.37 (1.30 – 3.94)	2.99 (1.81 – 4.63)	5.87 (4.22 – 7.90)
BA.2	0.29 (0.04 – 1.04)	0.52 (0.38 – 0.70)	0.66 (0.32 – 1.21)	0.30 (0.04 – 1.08)	2.19 (0.95 – 4.27)
BA.5	2.52 (0.69 – 6.32)	0.37 (0.14 – 0.81)	0.67 (0.55 – 0.80)	0,92 (0.65 – 1.27)	2,00 (1.14 – 3.22)
BQ.1	0.17 (0.05 – 0.43)	0.00 (0.00 – 1.70)	0.00 (0.00 – 4.86)	0,31 (0.18 – 0.51)	0.27 (0.01 – 1.51)
	% in-hospital deaths vs hospitalizations (90% CI)				
BA.1	7.99 (5.49 – 11.15)	12.36 (6.33 – 21.04)	13.95 (7.42 – 23.11)	13.16 (6.49 – 22.87)	17.69 (11.89 – 24.83)
BA.2	0.00 (0.00 – 8.22)	4.51 (2.96 – 6.53)	5.21 (1.71 – 11.74)	0.00 (0.00 – 4.56)	7.14 (2.36 – 15.89)
BA.5	20.00 (4.33 – 48.09)	1.71 (0.21 – 6.04)	3,41 (2.53 – 4.48)	4,11 (2.32 – 6.69)	7.83 (3.64 – 14.34)
BQ.1	0.59 (0.07 – 2.13)	0.00 (0.00 – 7.40)	0.00 (0.00 – 26.46)	1.24 (0.57 – 2.34)	1.19 (0.03 – 6.46)

Fisher exact test

To validate the observed disparities in disease severity according to vaccination status, we employed Fisher's exact test. This statistical tool is renowned for its utility in determining non-random associations between categorical variables, particularly with small sample sizes or uneven marginal distributions, and serves as a complementary approach to the previously described Pearson's χ^2 tests and the confident intervals. In fact, what we are looking for with Fisher is a quantitative test that reflects the same thing we see in Figure 2 of the main manuscript, i.e., that non-overlapping confidence intervals indicate a significant difference between two results. For ours, the Fisher's exact test confirms the relationship between different vaccination statuses and the severity of specific disease outcomes, thereby providing a statistically robust framework for our findings.

Table S4 presents the p-values obtained from the Fisher's exact test for the BA.1 variant, with respect to the percentages of cases and hospitalizations. In this example, we find no significant difference in outcomes between individuals who were vaccinated recently or up to six months ago. However, there are significant differences for those who were vaccinated more than six months ago or who were never vaccinated. In addition, Fisher's exact test shows no significant differences among hospitalized individuals who were vaccinated between six months and one year ago, but significant differences emerge when these individuals are compared with the unvaccinated. This pattern generally holds for other variants and measures, such as cases versus deaths or hospitalizations versus in-hospital deaths: recent vaccination yields more promising outcomes in terms of hospitalizations and deaths compared to those vaccinated more than six months ago, and especially when compared to those who have never been vaccinated. Additional tables and analyses are available in the **Supplementary Tables S3, S4, and S5** for all age groups.

Table S4 Results of the Fisher's exact test for hospitalization values relative to cases of the BA.1 variant, i.e. the first row of **Table S3** and the left-top points (pink symbols) in **Figure 2**.

	1-3 months	4-6 months	7-9 months	10+ months	No vaccination
1-3 months					
4-6 months	0.8064				
7-9 months	$\approx 10^{-12}$	$\approx 10^{-8}$			
10+ months	$\approx 10^{-8}$	$\approx 10^{-5}$	0.2055		
No vaccination	$\approx 10^{-34}$	$\approx 10^{-23}$	0.0014	$\approx 10^{-6}$	

Reduction in severity

Finally, to obtain Figure 3 in the main text, we have entered the term reduction in severity as 1-RR, where RR is the rate ratio entered earlier. Its calculation is straightforward, and we simply multiply by 100 to show the result as a percentage. Thus, in a visual way, the loss of vaccine efficacy can be contrasted by analyzing the confirmed cases of COVID-19, in quarterly terms, for the different age groups and the different variants. Its error is based on the propagation of standard errors, so we will obtain:

$$s_{\pm,1-RR} = 100\sqrt{\left(\epsilon_{\pm p,\mathrm{Vacc},i}/R_{\mathrm{Unvacc}}\right)^2 + \left(R_{\mathrm{Vacc},i}\epsilon_{\pm p,\mathrm{Unvacc}}/R_{\mathrm{Unvacc}}{}^2\right)^2}\,.$$

As we have done throughout this Suppl. Mat. Text S4, Table S5 finally shows the results for the term 1-RR, together with its errors corresponding to the efficacy against hospitalizations for the 80+ age group.

Table S5 Results for the $1 - RR$ term for hospitalizations vs positive cases according to the periods of each study variant for different vaccination statuses of individuals older than 80 years in Catalonia. The rest of the age groups can be found in **Suppl. Mat. Tables S3, S4, and S5**. Yellow background cells indicate that $p > 0.05$ when compared to the results of the same unvaccinated group.

		**1-3 months**	**4-6 months**	**7-9 months**	**10+ months**	**No vaccine**
BA.1	$100 \cdot (1 - RR_{\mathrm{Hosp}})$	71.2	70.4	32.5	44.5	Reference
	$s_{+,1-RR(\mathrm{Hosp})}$	5.3	8.0	17.7	15.4	
	$s_{-,1-RR(\mathrm{Hosp})}$	4.9	7.1	15.9	13.7	
BA.2	$100 \cdot (1 - RR_{\mathrm{Hosp}})$	67.6	63.5	67.0	38.2	Reference
	$s_{+,1-RR(\mathrm{Hosp})}$	13.1	8.9	10.3	20.0	
	$s_{-,1-RR(\mathrm{Hosp})}$	11.0	8.0	9.1	17.6	
BA.5	$100 \cdot (1 - RR_{\mathrm{Hosp}})$	34.2	49.0	43.6	36.7	Reference
	$s_{+,1-RR(\mathrm{Hosp})}$	41.2	13.4	10.7	13.3	
	$s_{-,1-RR(\mathrm{Hosp})}$	30.3	12.0	9.7	12.0	
BQ.1	$100 \cdot (1 - RR_{\mathrm{Hosp}})$	38.6	2.5	29.2	38.1	Reference
	$s_{+,1-RR(\mathrm{Hosp})}$	14.0	33.4	47.6	13.3	
	$s_{-,1-RR(\mathrm{Hosp})}$	12.7	29.6	35.4	12.1	

Suppl. Mat. Text S5. Severity of outcomes across age and vaccination status: expanded explanation and extended results

In the main manuscript, **Figure 2** shows all the ratios of hospitalizations to cases, mortality to cases, and mortality among hospitalized patients to total hospitalizations. Nonetheless, **Figure 2** makes a distinction at the points where the number of events is very small. For example, the 60-69 cohort aged is not included in the % Deaths vs Cases metric because there are only a few significantly comparable results in the number of deaths for variants BA.1, BA.2, and BA.5 in this age group. However, another example is the data for variant BA.5 in the first vaccination period (1-3 months), which is consistently marked due to its emergence in the spring-summer of 2022, more than 3 months after the first booster dose's extensive vaccination campaign in Catalonia at the end of 2021. Thus, significant results are mainly observed from the 4-6 months onward.

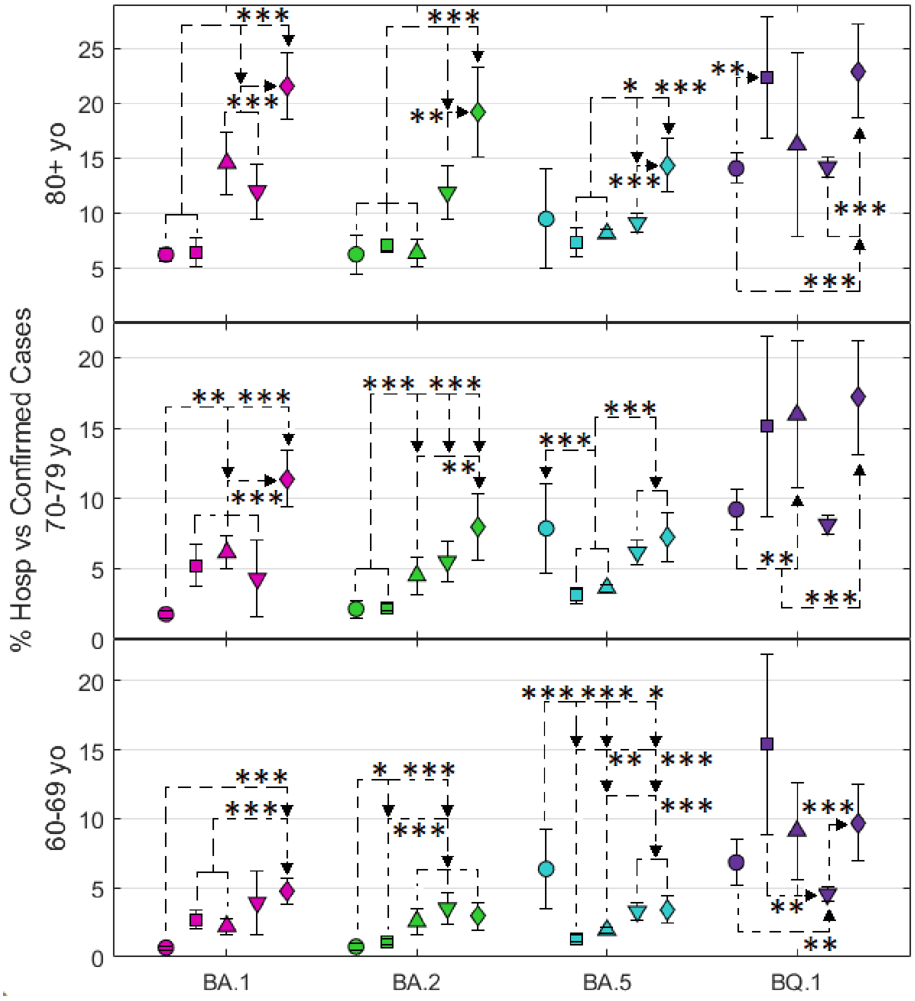

Figure S5 Plots displaying the percentage for hospitalizations relative to positive cases. All age groups and the four Omicron subvariants are shown. Symbols indicate different periods post-vaccination and non-vaccinated individuals. Error bars indicate 95% confidence interval. Dashed lines connecting different symbols with arrows highlight significant differences between the compared data points. Significance levels are denoted by asterisks: * corresponds to p-value < 0.05; ** to p-value < 0.01; and *** to p-value < 0.001.

Figure S5 show a similar representation of the **Figure 2**, but with arrows indicating significant differences that meet Fisher's test $p < 0.05$. We do this to facilitate the association and comparison of results from a single look at the figure. It should be noted, however, that since we have not made a priori a quantitative analysis of the minimum sample size, we may be making statistical errors. This seems the case at some points in the **Figure S5** with a large error bar, but still with

significant differences. A straight direct case is the 1-3 months post-vaccination of the BA.5, which has 100 times fewer cases than other results of BA.5, such as those vaccinated between 4-6 and 7-9 months (corresponding to the first campaign of booster dose against COVID-19 in Spain).

In **Figure S5** dashed arrows indicate significant differences. For example, focus on the hospitalizations for those aged >80 years for the BA.1 variant (pink symbols). The results for those vaccinated within 1-3 months (○) and 4-6 months (□) do not differ statistically from each other. The same is true for those vaccinated between 7-9 months (Δ) and 10+ months (∇). However, both vaccination periods (1-6 months and 7+ months) are statistically different from each other, and each is also significantly different from the results of the unvaccinated (◊).

Figures S6 and S7 show the same, but with deaths and in-hospital deaths for all ages, in contrast to **Figure 2**, which does so only for those over 80 years of age.

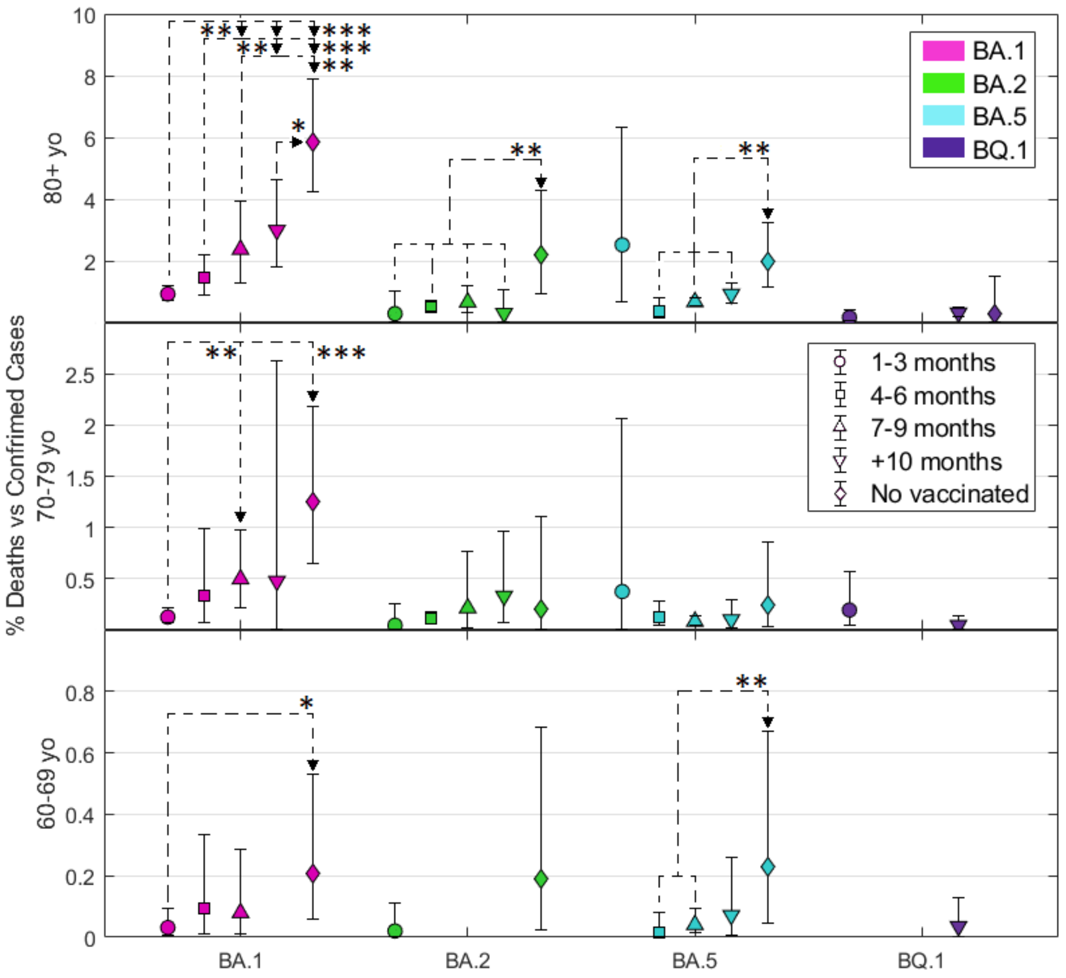

Figure S6 Plots displaying the percentage for deaths relative to positive cases. All age groups and the four Omicron subvariants are shown, nonetheless none BQ.1 is statistically different from each other. Symbols indicate different periods post-vaccination and non-vaccinated individuals. Error bars indicate 95% confidence interval. Dashed lines connecting different symbols with arrows highlight significant differences between the compared data points. Significance levels are denoted by asterisks: * corresponds to p-value < 0.05; ** to p-value < 0.01; and *** to p-value < 0.001.

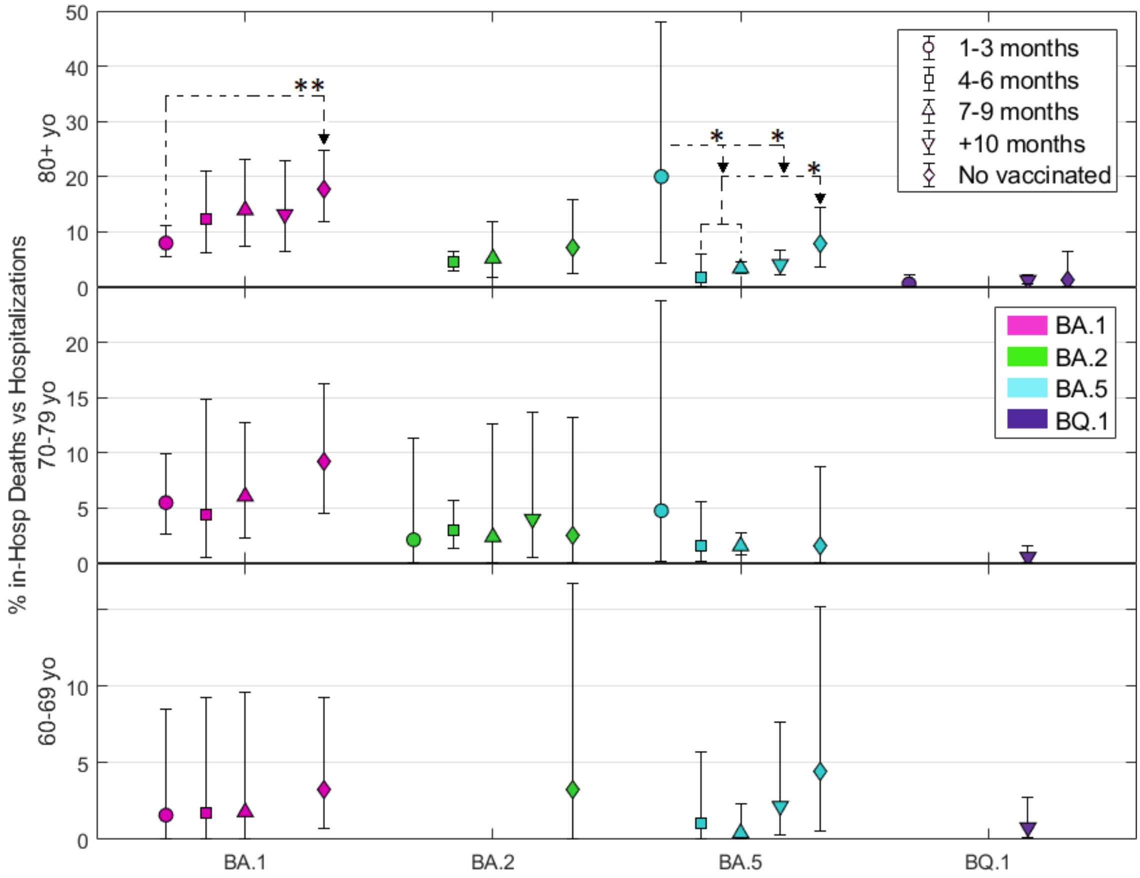

Figure S7 Plots showing the percentage of in-hospital deaths relative to hospitalizations. All age groups and the four Omicron subvariants are shown, but only BA.1 and BA.5 have some statistical differences between their results for the +80-age cohort. Symbols indicate different time periods after vaccination and unvaccinated individuals (see legend). Error bars show 95% confidence interval. Dashed lines connecting different symbols with arrows indicate significant differences between the compared data points. Significance levels are denoted by asterisks: * corresponds to p-value < 0.05; ** to p-value < 0.01; and *** to p-value < 0.001.

Figures S6 and S7 show some more significant results for 60-69 and 70-79 age cohorts in addition to the +80-age cohort of the main manuscript. Although there are not many results that add value to the discussion of the main article, we can highlight that, in general, the data indicate that the vaccine protects against the most severe cases and that, in the case of BA.1, there are significant differences between vaccination before or after 6 months of age. On the other hand, again, we obtain on one occasion in the case of those recently vaccinated for BA.5, although its trend with a trend not expected. This is the same case as the main manuscript, where very likely a false positive error is made.

To summarize the results, a **Figure S8** aggregates the percentages of the three-severity metrics evaluated – hospitalizations, mortality, and in-hospital mortality – across all Omicron variants combined throughout 2022. These consolidated results reiterate the significant impact of vaccination in reducing the severity of both hospitalizations and mortality across all age groups. The data underscore the efficacy of vaccination for all individuals >60 of age as it consistently correlates with reduced severity metrics, reinforcing the importance of vaccination campaigns in public health strategies against COVID-19.

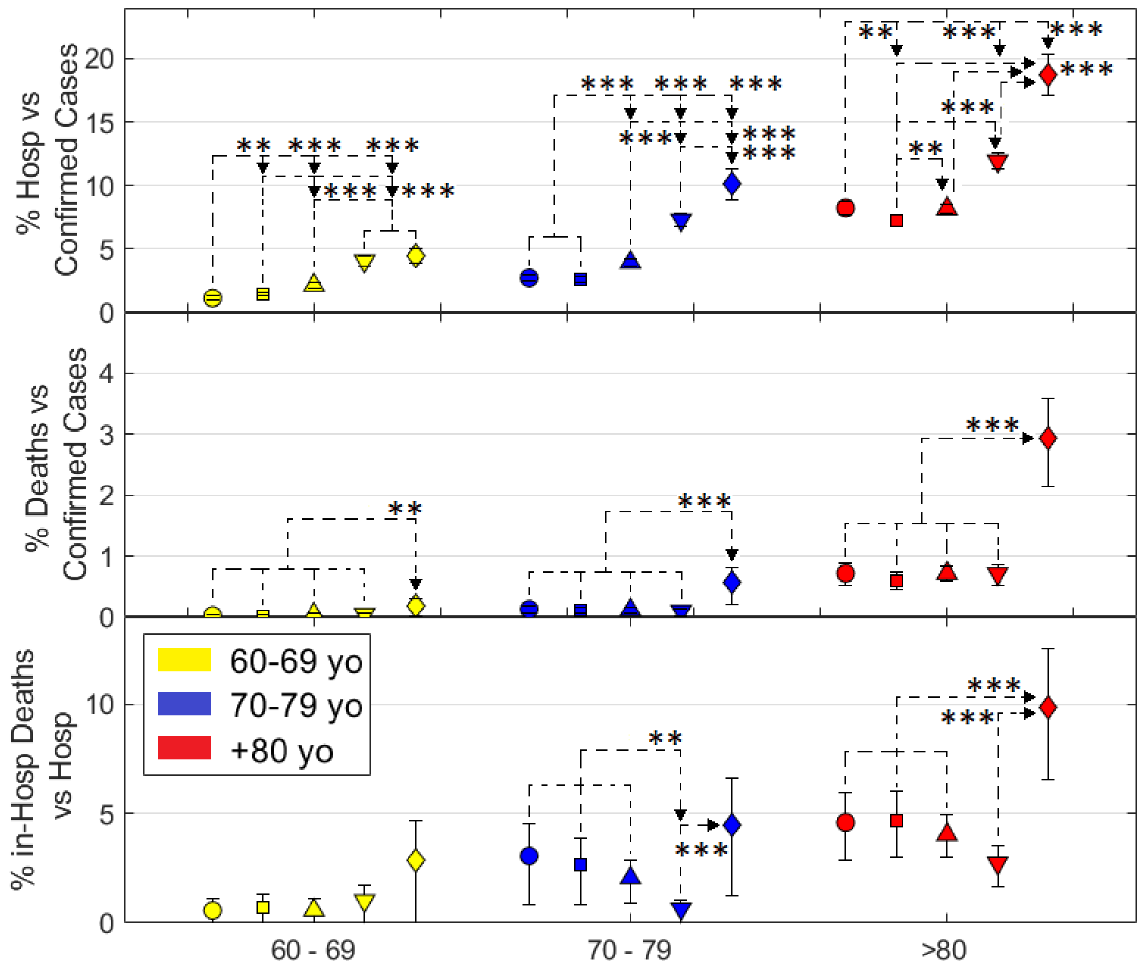

Figure S8 Percentage of hospitalizations (top) and deaths (middle) among COVID-19 cases and in-hospital deaths (bottom) among hospitalizations by age group and time since last vaccine dose. Age groups are represented by colour: blue for 60-69 years old, red for 70-79 years old, and yellow for 80+ years old. Data points are shown with different symbols for time intervals since last vaccine dose, including unvaccinated. Horizontal dashed lines indicate that these data points are not statistically different. Significance levels are denoted by asterisks: * corresponds to p-value < 0.05; ** to p-value < 0.01; and *** to p-value < 0.001.

Suppl. Mat. Text S6. Analysis of vaccine impact across age cohorts in 2022 and detailed mortality analysis data including deaths due to and with COVID-19

Finally, as a supplement to **Figure 3** in the main manuscript, we show the impact of the vaccine on severity reduction for the age groups 60-69, 70-79, and 80+ years in death versus confirmed cases. Although the inclusion of younger age cohorts results in a scarce large number of outcomes, it does not alter the discussion in the main manuscript. In fact, we do not add hospital deaths because they do not provide new information. The few new plotted results suggest that the protective effect of vaccination against severe outcomes remains important regardless of the categorization of mortality.

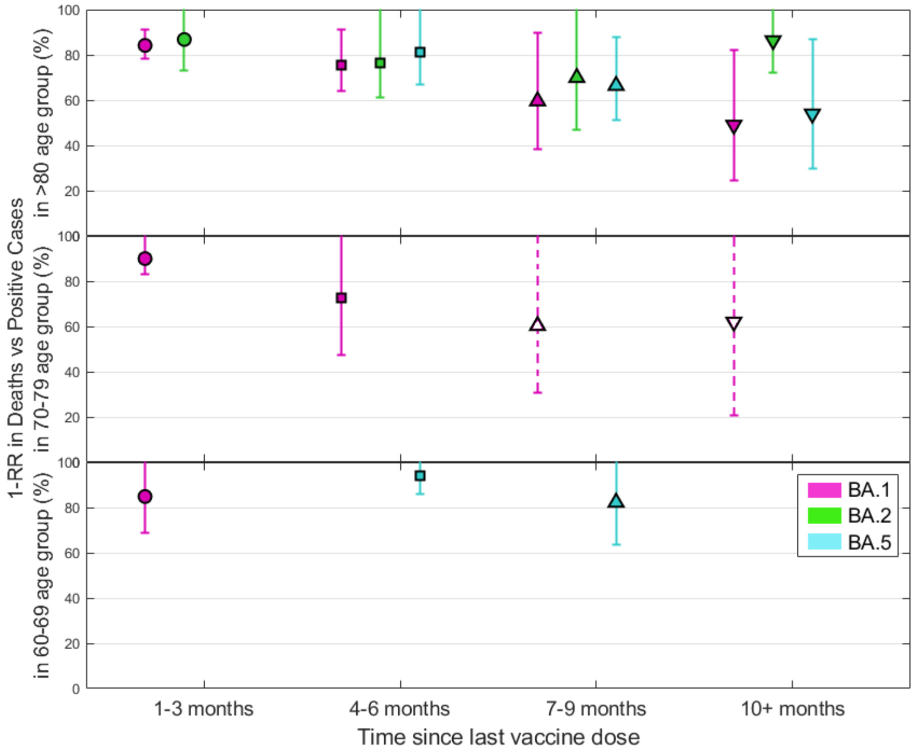

Figure S9 Reduction in severity of deaths from the Omicron subvariants BA.1 (pink), BA.2 (green), and BA.5 (blue) in all age cohorts of the study across different post-vaccination intervals. Solid symbols represent statistically significant values, while empty symbols indicate non-significant results compared to the unvaccinated group.

On the other hand, **Figure S10** presents the same analysis of the vaccine impact across all Omicron variants combined throughout 2022, similar to **Figure 3** in the main manuscript. The top panel shows the percentage reduction in hospitalizations, while the bottom panel shows the reduction in mortality. The data points are separated by age groups, including 60-69 years (blue points), 70-79 years (red), and those aged 80 years and older (yellow), highlighting the different efficacy of vaccines to reduce severe outcomes in these populations.

There is a clear trend for hospitalizations, indicating that vaccines are effective against severe disease outcomes, although decreases with time after vaccination. In particular, the decline in the vaccine impact is more pronounced at the 10+ month interval, highlighting the potential need for booster doses to maintain high levels of protection. However, the effectiveness of the vaccine in terms of deaths remains very constant for individuals aged 70 years and older. This implies that there is indeed a substantial difference between vaccination and non-vaccination, but there does not appear to be one for individuals vaccinated at different times since their last vaccination.

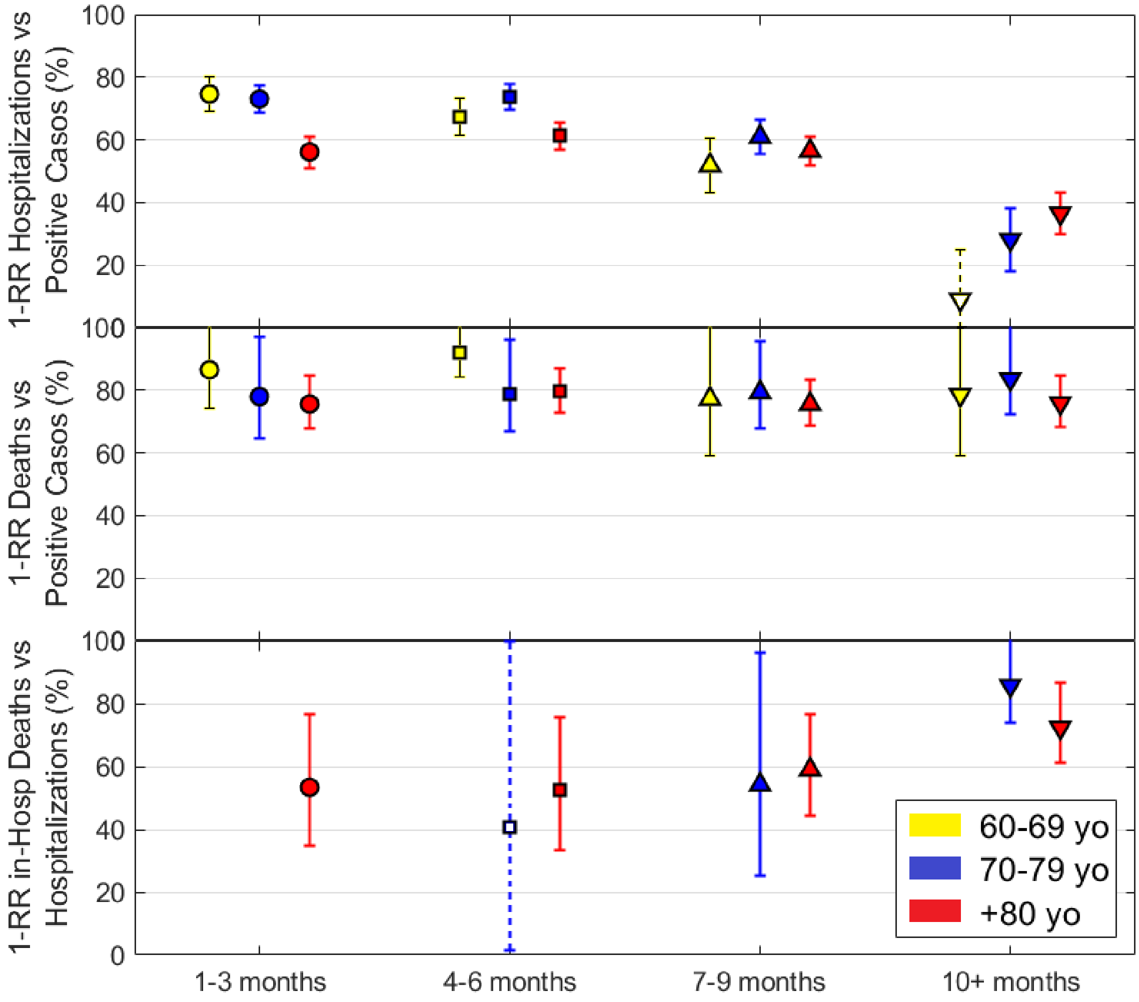

Figure S10 Reduction in severity (1-RR) in mitigating the number of hospitalizations and deaths for different age cohorts during the period that included the prevalence of the Omicron subvariants (BA.1, BA.2, BA.5, and BQ.1). This evaluation covers different time intervals after vaccination: 1-3 months (○), 4-6 months (□), 7-9 months (Δ), 10+ months (∇), and unvaccinated (◊).
